# Effect of the pH on the Antibacterial Potential and Cytotoxicity of Different Plasma-Activated Liquids

**DOI:** 10.3390/ijms232213893

**Published:** 2022-11-11

**Authors:** Aline da Graça Sampaio, William Chiappim, Noala Vicensoto Moreira Milhan, Benedito Botan Neto, Rodrigo Pessoa, Cristiane Yumi Koga-Ito

**Affiliations:** 1 Science Applied to Oral Health, Graduate Program of Institute of Science and Technology, UNESP, São José dos Campos 12245-000, Brazil; 2 Laboratory of Plasmas and Applications, Department of Physics, Faculty of Engineering and Sciences, São Paulo State University (UNESP), Guaratinguetá 12516-410, Brazil; 3 Plasmas and Processes Laboratory, Department of Physics, Technological Institute of Aeronautics, Praça Marechal Eduardo Gomes 50, São José dos Campos 12228-900, Brazil; 4 Department of Environment Engineering, Institute of Science and Technology, São Paulo State University (UNESP), São José dos Campos 12247-016, Brazil

**Keywords:** plasma-activated liquid (PAL), plasma-activated water (PAW), deionized water, distilled water, filtered water, saline, gliding arc discharge, *Escherichia coli*, antimicrobial effect, toxicity

## Abstract

In this study, different plasma-activated liquids were evaluated for their antimicrobial effects against *Escherichia coli*, as well as for their cytotoxicity on mammalian cells. The PALs were prepared from distilled (DIS), deionized (DI), filtered (FIL), and tap (TAP) water. Additionally, 0.9% NaCl saline solution (SAL) was plasma-activated. These PALs were prepared using 5 L/min air gliding arc plasma jet for up to 60.0 min of exposure. Subsequently, the physicochemical properties, such as, the oxidation-reduction potential (ORP), the pH, the conductivity, and the total dissolved solids (TDS) were characterized by a water multiparameter. The PALs obtained showed a drastic decrease in the pH with increasing plasma exposure time, in contrast, the conductivity and TDS increased. In a general trend, the UV-vis analyses identified a higher production of the following reactive species of nitrogen and oxygen (RONS), HNO_2_, H_2_O_2_, NO_3_^−^, and NO_2_^−^. Except for the plasma-activated filtered water (PAW-FIL), where there was a change in the position of NO_2_^−^ and NO_3_^−^ at some pHs, The higher production of HNO_2_ and H_2_O_2_-reactive species was observed at a low pH. Finally, the standardized suspensions of *Escherichia coli* were exposed to PAL for up to 60.0 min. The plasma-activated deionized water (PAW-DI pH 2.5), plasma-activated distilled water (PAW-DIS pH 2.5 and 3), and plasma-activated tap water (PAW-TAP 3.5) showed the best antimicrobial effects at exposure times of 3.0, 10.0, and 30.0 min, respectively. The MTT analysis demonstrated low toxicity of all of the PAL samples. Our results indicate that the plasma activation of different liquids using the gliding arc system can generate specific physicochemical conditions that produce excellent antibacterial effects for *E. coli* with a safe application, thus bringing future contributions to creating new antimicrobial protocols.

## 1. Introduction

The exposure of liquids to non-thermal atmospheric pressure plasmas (NTAPPs) generates plasma-activated liquids (PALs). This eco-friendly technology [1] is developed by the use of an ionized gas that is applied into or onto a liquid media [2,3,4]. Its activation leads to the production of reactive species generated from the gas phase of the plasma and the plasma–liquid interface interaction [5]. The reactive species dissolved in the liquid environment [3] have generated great scientific interest due to the proven actions of their active constituents in the environmental, agricultural, and health fields [6,7,8,9].

The NTAPP that are used in the activation of liquids promote the physicochemical reactions creating the reactive oxygen and nitrogen species (RONS), which are the main active constituents in PAL. The reactions involve the chemical radicals of hydrogen peroxide (H_2_O_2_), hydroxyl (OH), oxygen (O_2_), superoxide (O_2_^−^), ozone (O_3_), nitrates (NO_3_), nitrites (NO_2_), and peroxynitrites (ONOO^−^) [10,11,12,13,14,15,16]. Parameters such as the type of plasma generation gas, plasma power, reactor type, and the molecular interactions in the plasma–liquid interface can influence the composition, quantity, and lifetime of the RONS, in addition to changing the acidity of the activated solution [3,7,17].

Recently, many researchers have explored the potential applications of different types of plasma-activated water (PAW), such as distilled water [18,19], deionized water [20,21], and tap water [22,23,24]. Additionally, the activation of other liquids has been performed, such as buffered solutions (Phosphate-buffered saline—PBS), saline solutions [25], olive oil [26], sunflower oil [27], and radix arnebiae oil [28]. These liquids were tested both while they were pure or mixed with other substances [29,30] and have several applications in different fields, such as the microbial decontamination of water, plant cultivation, inducing a dormancy break in seeds, and the green irrigation of agriculture [20,31,32]. Their application in the food industry can also be highlighted due to the bacterial decontamination of food and food contact surfaces [31,32,33,34,35,36].

Another important field of investigation of PAL is in the health area. Due to the antimicrobial properties mainly attributed to the RONS, PAL has been used in different medical and dentistry fields [7,9]. Additionally, studies investigating the effect of PAL on wound healing and cancer treatment have also been carried out with promising results [7,9,18,31]. Due to the antimicrobial properties against bacteria, fungi, and virus [8,22,23,37], PAL have been used for the microbial disinfection and sterilization of medical and dental devices [7,9,38,39], and it has also been investigated for possible applications to infectious diseases [40,41].

The ram-negative bacterium *Escherichia coli* is a commensal inhabitant of the intestinal tract of humans and animals, and it is an indicator of fecal pollution. However, it presents pathogenic forms that can spread in wastewater, soil, and food [42,43]. *E. coli* is responsible for cases of mortality associated with outbreaks of severe gastrointestinal diseases which are transmitted by contaminated water and food [44,45] with increasing treatment difficulty due to their resistance to antibiotics [42,44,45,46]. Frieri et al. [46] pointed out the challenges in combating resistant bacterial infections leading to therapeutic limitations. In this context, PAL could emerge as a possible complementary treatment. In vivo studies have shown that exposure to PAL is safe and not toxic to tissues [47,48].

Therefore, in the literature, many studies show PAL’s effectiveness as an antimicrobial agent against *E. coli*. The antimicrobial properties of it open the way for the most diverse applications, ranging from the food area to water treatment, and its activation through the disinfection of wounds and hospital materials [9,18,32]. However, a detailed study of the physicochemical characterization of different liquids that are activated by NTAPP, namely, tap water, deionized water, distilled water, filtered water, and 0.9% NaCl saline solution, with the monitoring of the pH reduction and the generation of reactive species over time is necessary to elucidate how the transfer and generation of RONS affect the pH and the inactivation of *E. coli*. Another point to highlight in the present work is the investigation of cytotoxicity for mammalian cells of several types to activated liquids with different pHs and concentrations of RONS, which opens up the possibility of many direct applications and co-adjuvant treatments in humans. To achieve the proposed objectives, the experimental study was designed as follows: first, a plasma jet was generated in a forward vortex flow reactor (FVFR) gliding arc reactor using 5 L/min of compressed air, and the surfaces of the liquids were placed a few millimeters from the reactor nozzle.

After the activation, the liquids were characterized using a multiparameter meter to measure the pH, oxide-reduction potential (ORP), conductivity, and total dissolved solids (TDS). The RONS concentrations were estimated using a UV-Vis spectrophotometer. The antimicrobial properties of the PALs were evaluated against *E. coli* (ATCC 10799). Finally, their cytotoxicity to mammalian cells was evaluated.

## 2. Results and Discussions

### 2.1. Evaluation Physicochemical Properties of PALs

#### 2.1.1. Effect of Activation Time on Temperature and Volume of Activated Liquids

**Figure 1:**
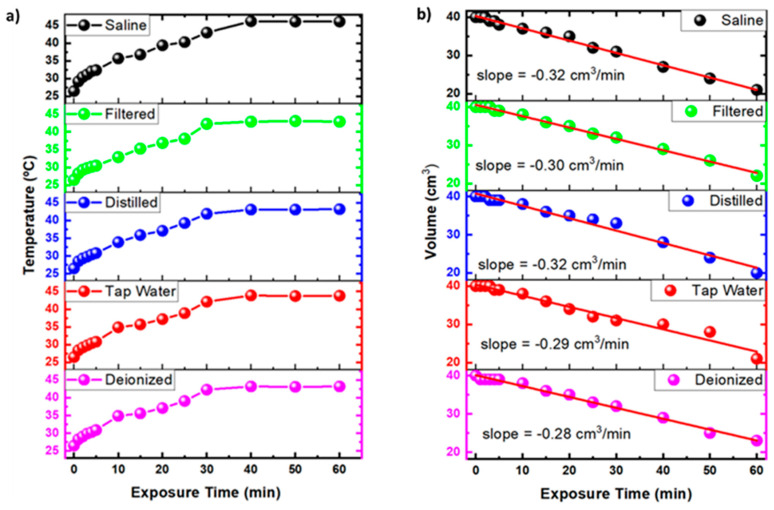
Treated liquid temperature (**a**) and liquid volume variation (**b**) as a functions of exposure time to non-thermal atmospheric pressure plasma (NTAPP) for saline solution, filtered water, distilled water, tap water, and deionized water.

Figure 1a shows the temperature variation over time during the plasma exposure. As observed, the temperature gradually rises between 25 and 40 °C until approximately 30.0 min of exposure to the NTAPP. After this period, the temperature stabilizes to a plateau of approximately 42 °C for the PAWs and 45 °C for the PAL-SAL. In contrast, for all of the activated liquids, there is a drastic reduction in the volume of the liquids with the time of exposure to the plasma (Figure 1b). This evaporation at such low temperatures is related to water corrosion by the sputtering due to the NTAPP [31]. The discharge time, liquid composition, plasma power, and plasma-liquid distance contribute to the evaporation [49]. The short-distance activation of the liquid increases the ionic wind speed, accelerating the sputtering, raising the evaporation, and increasing the concentration of the RONS [50]. The present work used a plasma jet which was generated by a forward vortex flow reactor (FVFR) gliding arc reactor using 5 L/min of compressed air. The broad interest in the gliding arc discharges results from the unusual chemical properties and enhanced reactivity of the heavy activated species (atoms, radicals, and excited molecules) that were produced in the plasma, which for the case of the air-based plasma allows there to be an increase in the RONS concentration [51].

#### 2.1.2. Effect of Activation Time on pH, ORP, and Conductivity

The physicochemical parameters of the PAL, such as pH, conductivity, ORP, and TDS, are characteristics that are influenced by the plasma generation system, the electrical and experimental configuration, the type, and the volume of activated liquid, working gas, and the reactor type [7,17,23,52]. Table 1 shows the parameters for PAW-TAP, PAW-DI, PAW-DIS, PAW-FIL, and PAL-SAL. Understanding these physicochemical parameters is paramount for standardizing the PALs, as the synergy between the plasma generation, plasma-liquid interaction, and biological application is increasingly important in the health sciences [7]. Table 1 shows the pH reduction of the activated PALs for up to 60.0 min. The plasma activation for 1.0 min showed a rapid drop in the pH values for all of the PALs, with a sharp decline for PAW-DIS and PAL-SAL from 6.10 ± 0.9 to 4.02 ± 0.9 and 7.20 ± 0.9 to 3.70 ± 0.9, respectively. After 5.0 min of activation, the pH values of approximately 3.50 were observed for all of the PALs, and they stayed within the standard deviation of ±0.9, except for PAW-DI, whose pH reached lower values in the order of 3.30 ± 0.9. The most significant pH changes were observed for PAW-FIL (4.94 ± 0.9 to 3.50 ± 0.9) and PAW-TAP (5.56 ± 0.9 to 3.55 ± 0.9). In contrast, there was a slight decrease in the pH for all of the PALs between 30.0 and 60.0 min (from 3.0 ± 0.9 to 2.5 ± 0.9); this behavior shows that the activation that takes place over time has more difficulty in reducing the pH of the liquids [7,10,23,53,54,55,56,57]. The ORP values increased significantly in ascending order for PAL-SAL, PAW-FIL, PAW-TAP, PAW-DIST, and PAW-DI after 60.0 min. Recent work has shown that the increase in the ORP with a reduced pH is influenced by the prolonged activation time and may be more effective at low activated volumes [23,54]. The increase in the conductivity values was also observed after the activation of the PALs over time. The same effect over time was observed by Schmidt et al. [53] and Lee et al. [57]. Ma et al. [33], Wu et al. [58], and Xiang et al. [59] who suggest that the increase in the conductivity is related to the presence of active ions that are generated in the plasma–liquid interaction process [33,58,60]. The 0.9% NaCl saline solution has a high conductivity (13,420 µS/cm), which after its activation for 60.0 min, has a value above the full scale of the multiparameter meter (>20,000 µS/cm). Schmidt et al. [53] reported a similar result. It is worth noting that the high conductivity is related to the presence of a large amount of sodium chloride (NaCl) particles that are dissolved in the activation process, where the initial TDS is 9320 ppm. After 60.0 min of exposure to the NTAPP, it reaches values of 17,400 ppm. The TDS increased for all of the PALs due to the reactive species that can dissolve the small particles in the liquids into smaller particles. Bourke et al. [61] and Zhou et al. [31] suggest that the physicochemical properties of the PALs, such as pH, ORP, and conductivity, influence their antimicrobial activity.

#### 2.1.3. Analysis of the Reactive Species of PALs

The production of PALs generates liquids that contain long-lived reactive oxygen and nitrogen species (RONS) such as nitrates (NO_3_^−^), nitrites (NO_2_^−^), nitrous acid (HNO_2_), and hydrogen peroxide (H_2_O_2_) and short-lived RONS such as hydroxyl (OH), nitric oxide (NO), superoxide (O_2_^−^), peroxynitrate (OONO_2_^−^), and peroxynitrite (ONOO^−^) radicals [24,31,62,63].

Table 2 shows the long-lived RONS and their concentrations in all of the investigated PALs. The generation and concentration of each RONS varied according to the pH value and the type of activated liquid, with there being an emphasis on the decreasing order: HNO_2_, H_2_O_2_, NO_3_^−^ and NO_2_^−^. Except for the plasma-activated filtered water (PAW-FIL), where there was a change in the position of NO_2_^−^ and NO_3_^−^ for some of the pHs. It is important to highlight that the data in Table 2 were extracted from the UV-Vis spectra according to Liu et al. [64] (for more details, see Section 3.3). As a baseline, an activation time of 1.0 min was used to monitor the variation of the RONS in mg/L. It can be observed for all of the PALs that after 1.0 min of activation, 9.3–23.7 mg/L of H_2_O_2_ was generated. After 60.0 min of activation, these values reached values from 86.6 mg/L (PAW-DIS) to 160.3 mg/L (PAW-DI) of H_2_O_2_. HNO_2_ was another RONS that exhibited continued growth during its exposure to NTAPP. Initially, after 1.0 min of activation, the concentrations in mg/L of HNO_2_ varied from 0.1 (PAW-DI) to 5.5 (PAW-SAL), however, after 60.0 min of activation, the concentrations had a variation of more than one hundred times ranging from 201.3 mg/L (PAW-DIS) to 525.0 mg/L (PAW-DI). In the case of NO_2_^−^, between 5.0 and 60.0 min of activation, there were no major changes in the NO_2_^−^ concentrations in mg/L, which is in agreement with the literature [64]. In contrast, for NO_3_^−^, a gradual increase in the concentration in mg/L was observed up to the exposure time of 30.0 min, and a drastic decrease occurred after 60.0 min of activation. This behavior was observed for all of the activated liquids. Therefore, we can verify that for the present work, only a direct relationship was found between the pH and the variation of HNO_2_ concentration. Due to the limitation of the method used (see Liu et al. [64]), it was not possible to detect some species, such as HOCl, which can be found in plasma-activated saline solutions. Indeed, Zeghoud et al. [65] reported that the reaction between the chloride and OH ions could result in the secondary radical HOCl. Mukhopadhyay and Ramaswamy [66] showed that a saline solution at low pH (2–3) could produce HOCl and HCl after an electrochemical oxidation.

In the microbial inactivation processes, high hydrogen peroxides and superoxide concentrations generate strong oxidizing properties [31]. In contrast, a more significant bactericidal effect can be attributed to O_3_ when it is compared to that of the RONS, namely H_2_O_2_ and NO_2_ [67]. However, the simultaneous associations among the reactive species produced in the different PALs is crucial to improve the antimicrobial effect of an activated liquid [68]. Recently, Zhou et al. [69] reported a high production of H_2_O_2_, NO_3_^−^, and NO_2_^−^ in PAW that was exposed to compressed plasma air in comparison to that of other gases.

The present work sought to increase the concentration of the RONS in the PALs by using compressed air gas as a source of plasma generation and using an FVFR-type reactor to generate a vortex plasma jet. As a result, high HNO_2_ and H_2_O_2_ levels were obtained in all of the samples. The samples PAW-DI and PAW-FIL at pH 2.5 had the highest percentage concentration of HNO_2_ and H_2_O_2_. NO_2_^−^ and NO_3_^−^ also appear in abundance in all of the PALs [70]. According to Burlica et al. [71] and Milhan et al. [9], the production of nitrite ions can arise from the dissociation of N_2_ and O_2_ during the plasma/air/water interaction, and these ions react with the H_2_O_2_, thereby generating the nitrate ions.

Therefore, in the present work, it was possible to obtain PALs with expressive concentrations of RONS that are of paramount importance in dental, biological, and medical applications.

### 2.2. Antimicrobial Effect of PALs on E. coli

**Figure 2:**
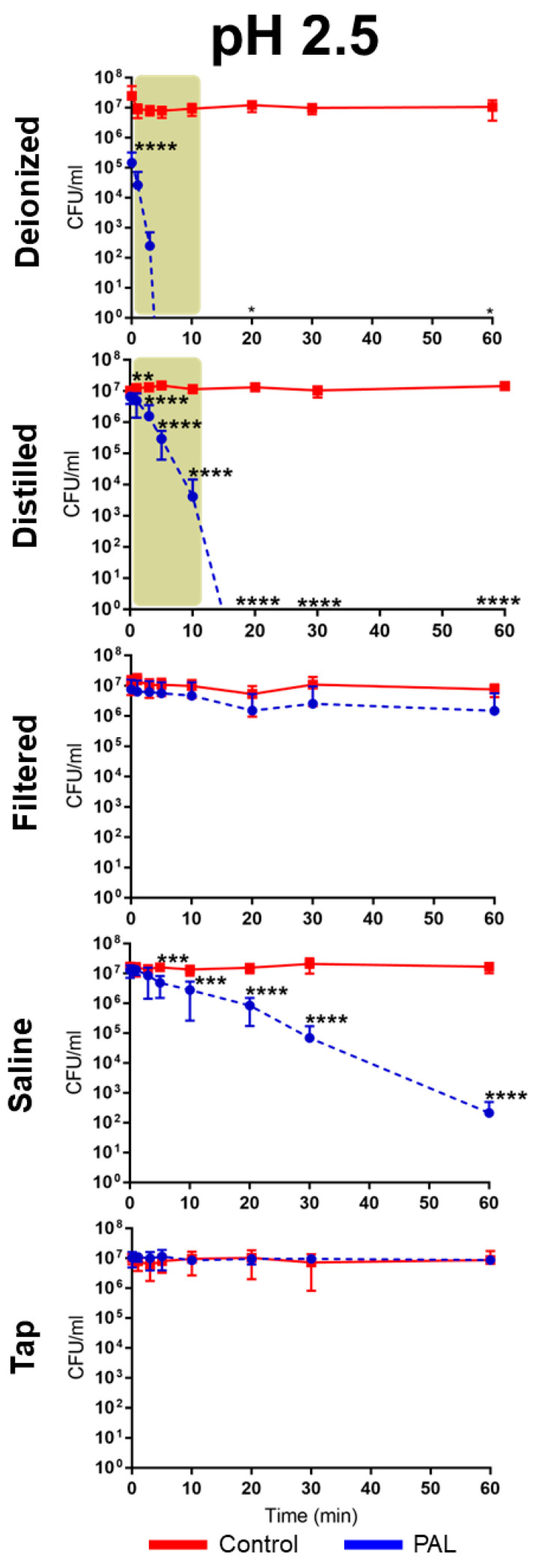
Overview of the antibacterial effects of *Escherichia coli* exposure in contact with different plasma-activated liquids over time at pH 2.5. The brown patterned area in graphic shows antimicrobial effect of *E. coli* at a short exposure time. (* *p* < 0.05; ** *p* < 0.01; *** *p* < 0.001; **** *p* < 0.0001 indicate statistical difference to untreated control group.)

**Figure 3:**
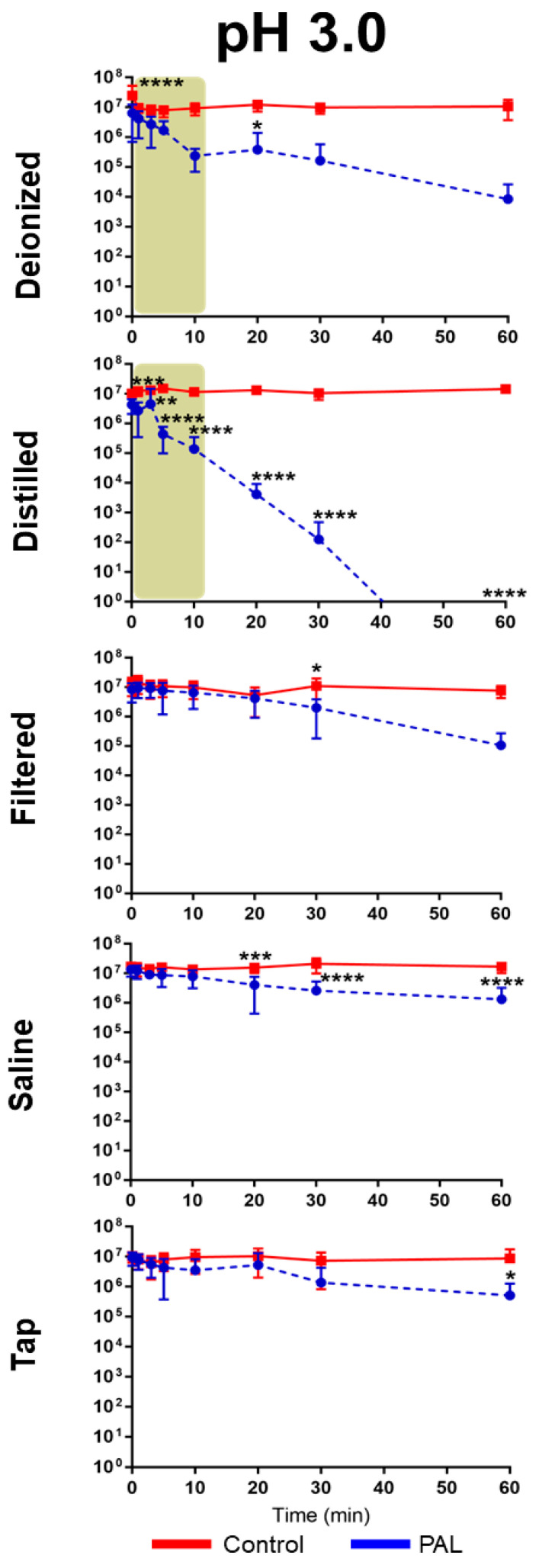
Overview of the antibacterial effects of *Escherichia coli* exposure in contact with different plasma-activated liquids over time at pH 3.0. The brown patterned area in graphic shows antimicrobial effect of *E. coli* at a short exposure time. (* *p* < 0.05; ** *p* < 0.01; *** *p* < 0.001; **** *p* < 0.0001 indicate statistical difference to untreated control group.)

**Figure 4:**
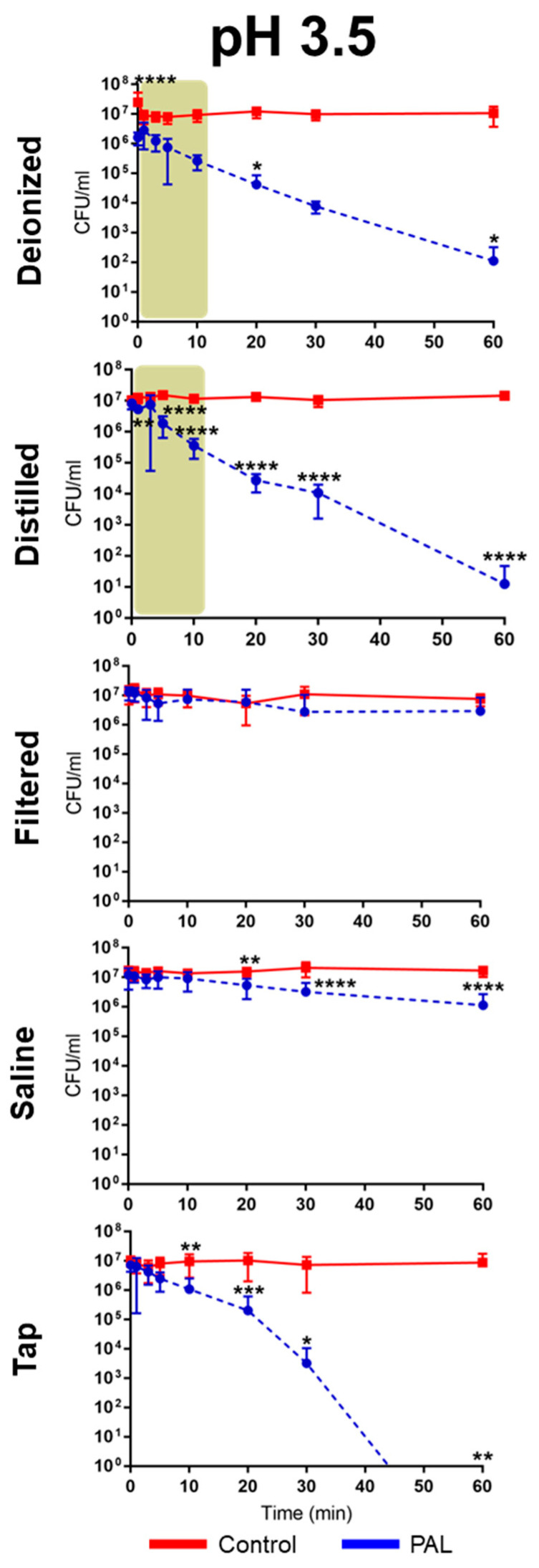
Overview of the antibacterial effects of *Escherichia coli* exposure in contact with different plasma-activated liquids over time at pH 3.5. The brown patterned area in graphic shows antimicrobial effect of *E. coli* at a short exposure time. (* *p* < 0.05; ** *p* < 0.01; *** *p* < 0.001; **** *p* < 0.0001 indicate statistical difference to untreated control group.)

The antibacterial activity of PALs with different pHs (2.5, 3.0 and 3.5) against *E. coli* is shown in Figure 2, Figure 3 and Figure 4. As can be seen, the bacterial reduction depends on the exposure time, which varies from 3.0 to 60.0 min, independent of the pH.

There was no count of viable microorganisms after 3.0 min of exposure to PAW-DI at pH 2.5 (Figure 2), with there being a significant reduction at immediate contact stage (*p* < 0.0001) and for 20.0 and 60.0 min timepoints (*p* < 0.05). The same result was observed after 10.0 min for PAW-DIS at pH 2.5 (*p* < 0.0001) and during the prolonged contact for PAW-DIS at pH 3.0 (*p* < 0.0001) and PAW-TAP at pH 3.5 (*p* < 0.05) after 30.0 min. After exposure for 60.0 min, a bacteriostatic effect was observed, with a significant reduction in the cell viability being detected in the PAW-DI, PAW-DIS (pH 3.5), and PAW-SAL (pH 2.5) groups of 6, 4, and 4 logs (*p* < 0.05, *p* < 0.0001 and *p* < 0.0001), respectively. However, there was a non-significant reduction by up to 3 logs for PAW -DI (pH 3.0). Exposure to PAW-TAP at pH 2.5 showed similar behavior to that of the control group. The treatments with PAW-FIL (all pHs), PAL-SAL (pH 3.0 and 3.5), PAW-TAP (pH 2.5 and pH 3.0) groups were the least effective, with there being a significant reduction for PAW-FIL (pH 3.0) at 30.0 min (*p* < 0.05) and PAW-SAL (pH 3.0 and 3.5) at 20 (*p* < 0.001 and *p* < 0.01), 30.0, and 60.0 min (*p* < 0.0001). Zhao et al. [72] observed a bactericidal effect on *E. coli* with PAW-DI (pH 2.5) that was activated (stored at 4 °C) by the plasma jet with air gas after 10.0 min of exposure. In contrast, bactericidal activity in *E. coli* by PAW-DIS in the DBD plasma of the air gas at about pH 3.0 and 2.7 after 30.0 min of contact time was observed with a 5-log reduction in it [73]. However, a bacteriostatic effect of the same reduction was observed by Chiappim et al. [23] in *E. coli* that was exposed for 30.0 min to PAW-TAP (pH 3.5) that was activated by the plasma gliding arc of air gas. The PAW-FIL and PAW-SAL in our study showed a lower antibacterial effect. The impact of filtered waters (carbon purification and reverse osmosis) that were activated by surface barrier discharge (SDB) from different regions was investigated by Simon et al. [56]. This study demonstrated that the chemical composition of the water source in each region could influence the antimicrobial activity. An investigation of PAL-SAL at pH 3.75–2.8 by DBD air gas plasma did not obtain an antimicrobial effect after 60.0 min of contact time [73]. It is important to highlight that although PAW-SAL has a high ORP, conductivity, and TDS (Table 1), the antibacterial activity is low. According to Kondeti et al. [72], the antibacterial activity is related to the higher pH in a saline solution, which increases the concentration of long-lived species, namely, H_2_O_2_, and HOCl [72]. In contrast, in our work, the pH is low (between 2.5 and 3.5), and consequently, the concentration of ClO^−^ is low and probably, there is a decrease in the generation of HOCl.

**Figure 5:**
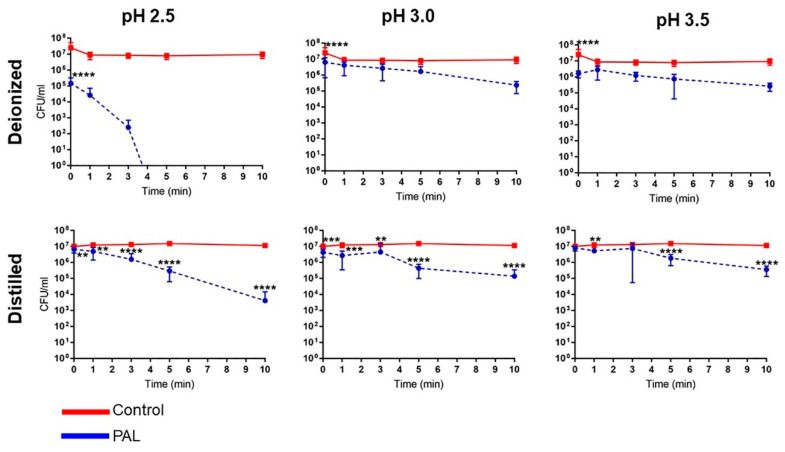
The expanded vision of the antibacterial action of *Escherichia coli* a short exposure time under plasma-activated distilled (PAW-DIST) and deionized water (PAW-DI). (** *p* < 0.01; *** *p* < 0.001; **** *p* < 0.0001 indicate statistical difference to untreated control group.)

Our results showed that bacterial inactivation kinetics depended on the PAL and the microbial exposure time, which is similar to that which was previously observed by Schmidt et al. [53]. In the literature, different start times of the antibacterial action (*E. coli*) in contact with other PALs were also observed [23,53,74], and the association of the effect by species such as H_2_O_2_, NO_2_^−^, and NO_3_^−^ is recurrent in the literature [59,69,72,73,74]. Our study detected high concentrations of HNO_2_, and H_2_O_2_, NO_2_^−^ and NO_3_^−^, and moderate concentrations of the radicals for the different PALs. PAW-DI and PAW-DIS at pH 2.5 presented an effective activity against *E. coli* after a short time (3.0–10.0 min; Figure 5) when they were compared to the other liquids that were tested. The influence of the antimicrobial action of the PALs at a low pH is suggested by [10]. The highest production of RONS with intense bactericidal activity was observed at a low pH level. However, with a significant dependence on the liquid that was used. It is suggested that the target of action of RONS in Gram-negative bacteria occurs in the cell wall. In gram-positive bacteria, it acts on the intracellular components, causing severe damage that can lead to cell death [72]. Oxidative stress that is generated by the action of the reactive species [75] as well as the accumulation of RONS around the membrane [76] can damage the cell membrane integrity, causing structural and functional damage to the cell structures, with disruption and morphological changes occurring, in addition to the damage and leakage of the proteins and genetic components (DNA and RNA), leading to microbial inactivation. [35,75,76]. Wu et al. [58] reported that the combination of H_2_O_2_, and the reactive species of OH and NO radicals contribute to bacterial lethality. However, changes to the cell wall of *E. coli* have been reported after PAW’s contact with a plasma jet of pure N_2_ gas, which was humidified with H_2_O and HNO_3_ steam, and a GAPJ system with air gas [55,59,74]. Gram-negative species are more sensitive to PALs [53,55]; this effect is related to the thinner cell structure in these species [77,78]. Corti et al. [79] also reported the influence of the growth phase of *E. coli* during the plasma treatment, and [72] the effect of PAW depends on the bacterial species. Therefore, future studies on the different growth stages of gram-positive and -negative pathogens should be investigated.

### 2.3. Cytotoxicity Test

**Figure 6:**
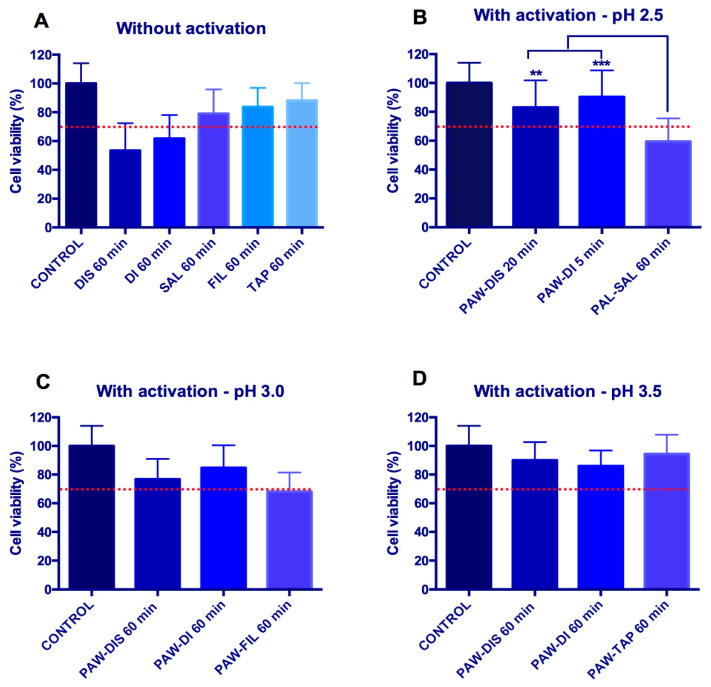
Graphics showing the percentage of viable cells obtained with MTT assay. (**A**) Liquids without plasma activation. (**B**–**D**) Liquids with plasma activation. The most antimicrobial liquids were evaluated in the shortest exposure time for pH = 2.5 (**B**), pH = 3.0 (**C**) and pH = 3.5 (**D**). The dashed line represents the normative value of cell viability (70%). (** *p* < 0.01; *** *p* < 0.001 indicate statistical differences.)

Figure 6 shows the percentage of viable cells in the groups after their exposure to non-activated and activated liquids. The exposure of the cells to non-treated distilled water and deionized water for 60.0 min shows values that are below the normative limit of cell viability (70%), however, the non-treated filtered and tap water were not cytotoxic. This result probably is related to the hypotonic stress that is caused by DIS and DI water that induces cell swelling and may cause cell death through a caspase and mitochondria-dependent mechanism [80]. This event is mostly observed in cancer cells, although a reduction in the cell viability may also occur with normal cells after the transition of them from a hypertonic to a hypotonic solution [80,81].

Interestingly, the activation of distilled and deionized water increased the cell viability. In the condition with pH 2.5, more than 80% of the viable cells were observed after their exposure to PAW-DIS for 20.0 min and PAW-DI for 5 min. Similarly, the exposure of the cells to PAW-DIS for 60.0 min and PAW-DI for 60.0 min did not induce a cytotoxic effect at pH 3.0 or 3.5. The activation of DIS and DI water generates the production of different reactive species, specially H_2_O_2_ and HNO_2_, which could have acted in the reduction of the hypotonic stress of these two waters. Future studies will be required to understand the possible underlying mechanisms related to this positive event.

On the other hand, the activation of saline solution reduced the cell viability to 59% after 60.0 min of exposure at pH 2.5. The cell viability value of this group was statistically different from the others that were evaluated at this same pH (PAW-DIS for 20.0 min and PAW-DI for 5.0 min) (*p* < 0.01, ANOVA Test). The plasma activation of saline probably resulted in an HOCl increase. This compound is unstable in acidic conditions, and the concentration of ClO^−^ decreased according to [82]. Moreover, the PAL-SAL that was activated in our study probably had an HOCl concentration. Doses of HOCl have been associated with the necrosis and apoptosis of mammalian cells, while growth arrest may occur with lower amounts of this compound [83]. In this way, our findings suggested that in the conditions of this study, the plasma-activated saline does not seem to be as promising as an antimicrobial agent, such as the PAWs.

No statistical difference was observed between the different types of the PAWs at pHs from 2.5 to 3.5 (*p* > 0.05, ANOVA and Kruskal–Wallis Tests) (Figure 6), which produced means that were approximately at the same level or higher than the normative limit of cell viability. Similarly, a previous study demonstrated that a deionized PAW that was generated by a microwave plasma system is not cytotoxic to keratinocytes or fibroblasts after 24 h of exposure [57]. In addition, a long-term in vivo study carried out in mice observed that 60 days, the PAW ingestion led to no body change in the structural and physiological integrity of the organs [84]. Additionally, another in vivo study showed that no acute toxicity was detected in rabbits after 30 days after a bone marrow injection with different PALs. Low amounts of PBS, saline, and culture medium that were activated by the plasma, with a near neutral pH, was administered to the animals. No unusual pathological changes in the organs or blood alterations were observed [47].

A rapid antimicrobial activity, a non-toxic property, water solubility, an active solution concentration, and a sustainable production are ideal properties for antiseptic and disinfectant solutions [85,86]. In our study, the short and long-term bactericidal action against *E. coli* by different PAWs, with the absence of toxicity effects, indicates that PAWs could be useful for different clinical applications, mostly the ones that are related to gastrointestinal diseases that are commonly caused by this bacterium [87]. This applicability could encompass the irrigation of surgical sites in appendicitis surgeries [88] or laparoscopic lavage, which has been useful for perforated diverticulitis [89]. Considering that *E. coli* is often related to different enteric diseases, such as diarrhea or dysentery [88], the treatment of drinking water may also represent a possible treatment. For this, future in vivo studies that evaluate the plasma-activated liquids locally and systemically at different pHs and times of exposition should be performed.

## 3. Materials and Methods

### 3.1. Preparation of PALs

**Figure 7:**
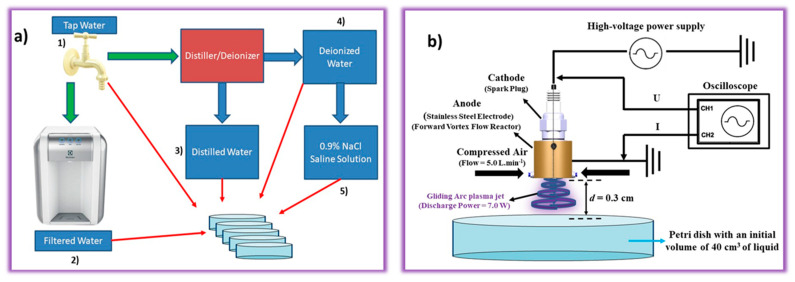
(**a**) Schematic illustration of the liquids used in the present work (tap water, filtered water, distilled water, deionized water, and 0.9% NaCl saline solution; (**b**) plasma activation experimental setup.

In the present work, different liquids were activated by NTAPP. Figure 7a shows the different liquids that are included in this study, namely, (1) tap water, (2) filtered water, (3) distilled water, (4) deionized water, and (5) 0.9% NaCl saline solution.

Tap water was the primary source, and this was collected at São José dos Campos, São Paulo, Brazil (23°12031.2″ S 45°52040.9″ W) [22]. The tap water was filtered in a water purifier (PE11B, Electrolux do Brazil S/A, Manaus, AM, Brazil) through a first filtration step with active carbon (suppressing odors and tastes) and a second filtration step with a polypropylene filter to remove the particles, and after this process, the filtered water which was used in work was obtained. The distilled water was obtained when the tap water was subjected to the distillation process (Purelab Option-Q, Elga labwater). The deionized water was processed in the same device that was used for the distillation, so the deionized water was tap water that has been distilled and then, deionized. Finally, the 0.9% NaCl saline solution was prepared by adding 9.0 g of NaCl to one liter of deionized water.

It is worth mentioning that the choice of different liquids is of paramount importance to understand the activation mechanism of NTAPP and to verify how the antimicrobial effect changes in different activated liquids using the same plasma parameters, such as power, frequency, distance, voltage, and electric current. Figure 7b shows the preparation of the plasma-activated liquids (PALs). The system used was a gliding arc plasma jet (GAPJ) that was generated in a forward vortex flow reactor (FVFR) with a compressed air flow of 5 L min^−1^ (Schulz CSD 9/50, Joinville, SC, Brazil). Basically, FVFR produces a gliding arc plasma jet (GAPJ) between the cathode (spark plug) and an anode which was made of stainless steel and designed in a cylindrical shape. Therefore, the experimental setup comprises a plasma reactor, a high-voltage power supply (Arternis 0215 model, Inergiae, Florianópolis, SC, Brazil) operating at a frequency of 19.2 kHz, an oscilloscope (Keysight DSOX1202A, Keysight, Santa Rosa, CA, USA), a high voltage probe (Tektronix P6015A, Tektronix, Beaverton, OR, USA), a self-adjusting current probe (Agilent N2869B, Agilent, Santa Clara, CA, USA), and a Petri dish with liquid. In the activation step, volumes of 40 cm^3^ of tap water, filtered water, distilled water, deionized water, and 0.9% NaCl saline solution were used, which were individually placed 0.3 cm from the nozzle of the GAPJ reactor, and the activation times investigated were 5.0, 30.0, and 60 min at pH 3.5, 3.0, and 2.5, respectively. After the activation, the samples received the following denomination: PAW-TAP (activated tap water), PAW-DI (activated deionized water), PAW-DIS (activated distilled water), PAW-FIL (activated filtered water), and PAL-SALT (0.9% activated NaCl saline solution).

### 3.2. Characterization of the Gliding Arc Plasma Jet

**Figure 8:**
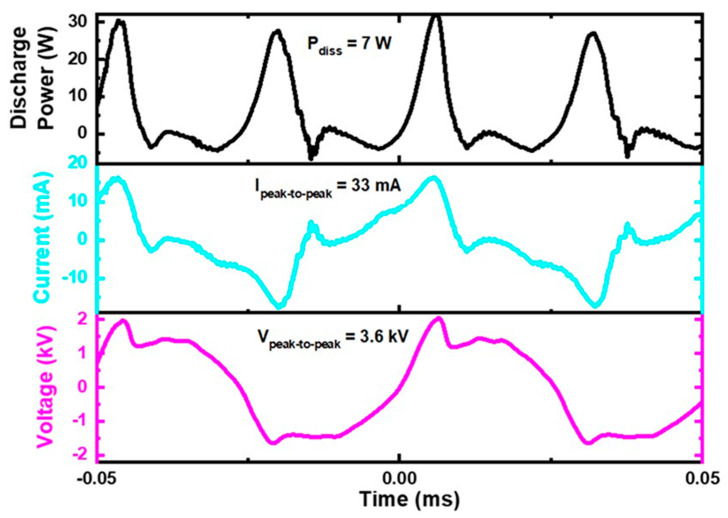
Discharge power, voltage, and current waveforms of GAPJ operating with the flow of 5 L min^−1^ (compressed air) and frequency of 19.2 kHz. All the electrical parameters were carried out while the plasma and liquid were in contact.

The voltage and current waveforms of the GAPJ discharge in contact with the liquids were obtained from the experimental setup (Figure 8) using a high voltage probe and a self-adjusting current probe. All of the electrical signals were recorded using a digital oscilloscope, and the current signal was obtained directly using the grounded electrode. The waveforms, voltage, electrical current, and power were unchanged for the different liquids. Figure 8 shows the discharge power, voltage, and current characteristic curves as a function of time. The electrical power that dissipated into the plasma was obtained according to [22]. The electrical parameters were: (i) the peak-to-peak voltage of 3.6 kV, (ii) the peak-to-peak current of 33 mA, and (iii) an electrical power that dissipated into the plasma of 7 W. The optical emission spectroscopy was carried out in our previous study [22].

### 3.3. Characterization of the Temperature, Volume, and Physicochemical Parameters of PALs

The in situ PAL was characterized in terms of the temperature using thermal images of the liquids during its activation using an IR camera (model TiS 10, Fluke, Everett, WA, USA). The measurement of the volume variation during the activation was carried out using a graduated cylinder. The ex situ PAL was characterized in terms of physicochemical parameters, namely, pH, ORP, TDS, and conductivity using a multiparameter meter (Combo 5, Akso, São Leopoldo, RS, Brazil), and a UV-Vis spectrophotometer (Evolution 201, Thermo Scientific, Waltham, MA, USA) was used to detect the presence of the reactive oxygen-nitrogen species (RONS). The optical absorbance measurements in the UV-Vis region (190–900 nm) were characterized by an instrumental accuracy of 0.001. The UV-Vis spectrophotometer was set up with a spectral resolution of 0.2 nm at a scan speed of 120 nm/min. The samples were contained in a square quartz cuvette of 3.5 mL (K22-135Q, Kasvi, São José dos Pinhais, SP, Brazil) and had a standard optical path of 10 mm with two polished sides. A background curve was obtained using an empty quartz cuvette to get the UV-Vis spectrum. Subsequently, a quartz cuvette containing 3.5 mL of the liquids was used to generate the baseline. In this way, it is possible to guarantee that the intensities in the UV-Vis spectrum of the PALs are related to the reactive oxygen-nitrogen species (RONS). After obtaining the baseline (liquids spectrum), the cuvettes were cleaned, the aliquots of the PAL were added (pH 2.5, 3.0, or 3.5), and each PAL’s relative UV absorption spectrum was obtained.

#### Absolute Concentrations of H_2_O_2_, NO_2_^−^, NO_3_^−^, and HNO_2_

**Figure 9:**
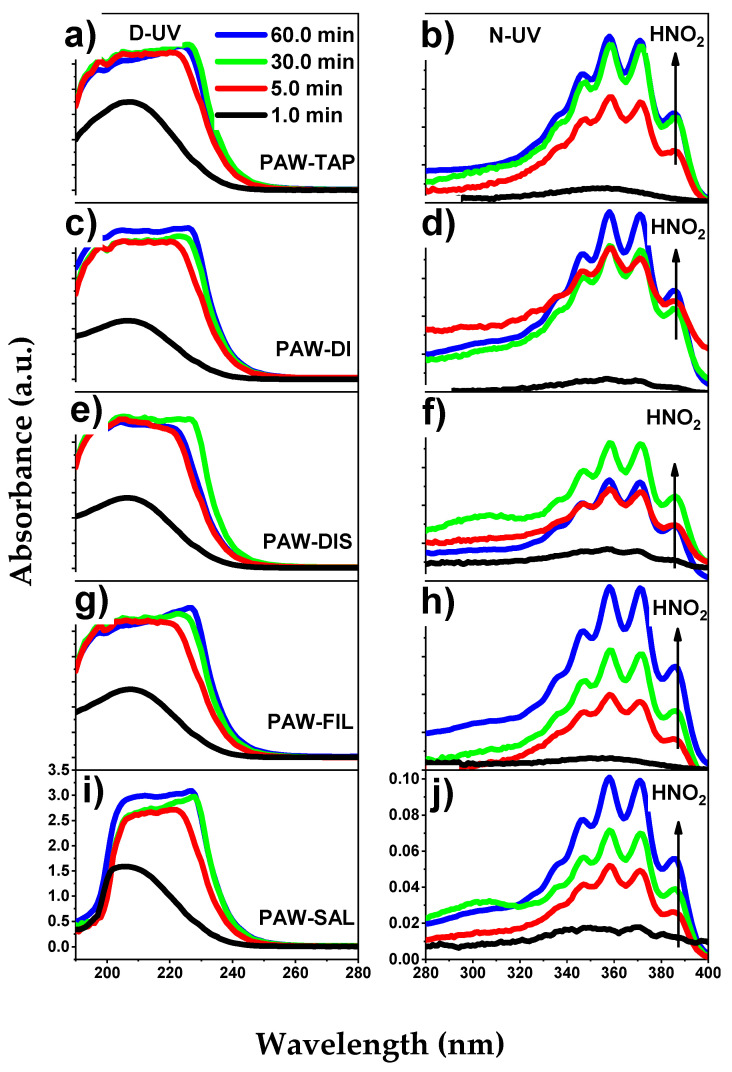
UV absorption spectra of activated liquids for 1.0, 5.0, 30.0, and 60.0 min. (**a**,**c**,**e**,**g**,**i**) represent the deep UV absorption spectra of tap water, deionized water, distilled water, filtered water, and 0.9% saline solution that were activated. (**b**,**d**,**f**,**h**,**j**) represent the near UV absorption spectra of tap water, deionized water, distilled water, filtered water, and 0.9% saline solution that were activated with the pattern of HNO_2_ formation.

The present work uses the technique that was developed by Liu et al. [64] and which was initially introduced by Oh et al. [63] to obtain the concentrations of H_2_O_2_, NO_3_^−^, NO_2_^−^, and HNO_2_. The purpose of using this technique is to avoid significant errors in the calculated RONS concentration because the absorption lines of H_2_O_2_, NO_3_^−^, and NO_2_^−^ overlap between 190 and 230 nm [64]. The UV-Vis spectra that are shown in Figure 9 were used. Additionally, with the help of Equation (1), the Matrix (2) was set up, and they were solved simultaneously to obtain the H_2_O_2_, NO_3_^−^, and NO_2_^−^ concentration in mg/L.

(1) $$A_{\lambda }=\alpha \left[{\mathrm{NO}}_{3}^{-}\right]+\beta \left[{\mathrm{NO}}_{2}^{-}\right]+\varepsilon \left[H_{2}O_{2}\right]\;$$

(2) $$\left(\begin{array}{c} \left[{\mathrm{NO}}_{3}^{-}\right]\\ \left[{\mathrm{NO}}_{2}^{-}\right]\\ \left[H_{2}O_{2}\right] \end{array}\right)={\left(\begin{array}{ccc} \alpha \left(\lambda_{1}\right) & \beta \left(\lambda_{1}\right) & \varepsilon \left(\lambda_{1}\right)\\ \alpha \left(\lambda_{2}\right) & \beta \left(\lambda_{2}\right) & \varepsilon \left(\lambda_{2}\right)\\ \alpha \left(\lambda_{3}\right) & \beta \left(\lambda_{3}\right) & \varepsilon \left(\lambda_{3}\right) \end{array}\right)}^{-1}\left(\begin{array}{c} A_{\lambda 1}\\ A_{\lambda 2}\\ A_{\lambda 3} \end{array}\right)/l$$

where α, β, and ε are the molar absorptivity coefficients and l is the optical path length.

The simultaneous solution was performed for wavelengths which were defined at 230.0, 235.0, and 250.0 nm, according to [64]. With the aid of Equation (3), the concentration of nitrous acid (HNO_2_) was found because according to Tachibana et al. [90], the composition of nitrous acid (HNO_2_) and its ion (NO_2_^−^) depends on the level of the pH in the solution with the acidity constant of HNO_2_ being pKa=3.38 at room temperature. As seen in the near UV (N-UV) region from 280 to 400 nm (Figure 9b,d,f,h,j), the HNO_2_ absorption spectra begin to appear when the pH drops dramatically. In the case of the present reactor, considerable concentrations of HNO_2_ are observed after 5.0 min of activation (see Table 2). This shows a direct relationship between a decreased pH and an increased in nitrous acid (as show in Equation (3)).

(3) $$\left[{\mathrm{HNO}}_{2}\right]=\frac{\left[{\mathrm{NO}}_{2}^{-}\right]}{10^{\mathrm{pH}-\mathrm{pKa}}}$$

### 3.4. Antimicrobial Effect of PALs on Escherichia coli

The reference strains of *E. coli* (ATCC 10799) which were stored at −80 °C were cultivated in a fresh culture medium containing Brain Heart Infusion (BHI) agar. The methodology described in [72] was adopted with modifications. A standardized suspension containing 10^7^ cells/mL of *E. coli* was prepared in a sterile saline solution (NaCl 0.9%) with the aid of a spectrophotometer (AJX-1600, Micronal, São Paulo, SP, Brazil), according to the following parameters: wavelength (*λ*) of 600 nm and optical density (O.D.) of 0.019. The aliquots of 1 mL of inoculum were exposed at 4 mL of plasma-activated liquids (PALs), which was followed by their analysis at timepoints of 0.0, 1.0, 3.0, 5.0, 10.0, 20.0, 30.0, and 60.0 min. The PALs with different pHs (2.5, 3.0 and 3.5) were tested. The bacterial reduction was evaluated by using serial dilution and plating on BHI agar, which was followed by their incubation at 37 °C for 24 h. For the controls, non-activated liquids were used. The data were obtained in 4 replicates at two independent events.

### 3.5. Cytotoxicity Evaluation of PALs

Oral keratinocytes (NOK) were grown in Dulbecco’s Modified Eagle’s medium (DMEM), supplemented with 10% fetal bovine serum and 1% penicillin (100 U/mL)/streptomycin (100 mg/mL). The cells were maintained at 37 °C in 5% CO_2_. For the assay, the cells were seeded at a density of 8 × 10^3^ cells/well in 96-well plates, which were then incubated for 24 h for the cell adhesion. After this period, the cells were exposed to the most antimicrobial PALs for each pH, and these were standardized as the ones that led to highest bacterial reduction in the shortest time of exposure. The proportion of the cell culture medium and the PALs was 1/1 (100 µL of each solution). The non-activated liquids were also analyzed using the same methodology. After their incubation for 5.0, 20.0, or 60.0 min, the cells were washed with Hanks’ Balanced Salt Solution (HBSS) and re-incubated for 24 h.

To measure the cell viability, 100 µL of 3-(4,5-Dimethylthiazol-2-yl)-2,5-diphenyl tetrazolium bromide (MTT) was added to each well, and the plates were incubated for 1.0 h. After this, the formazan crystals were dissolved with Dimethyl Sulfoxide (DMSO). The resulting optical density of the solution was measured using a spectrophotometer at 570 nm. The absorbance data were normalized to the untreated control group (=100%). Six replicates were used in two independent experiments (*n* = 12). The samples presenting a cell viability that was below 70% were considered to be cytotoxic [91].

### 3.6. Statistical Analyses

The results of the antimicrobial activity and cell viability tests were analyzed by One-way ANOVA using the Graphpad Prism 7.0 software (Graphpad Software Inc., San Diego, CA, USA), which was followed by multiple comparison analyses such as Tukey’s test and Kruskal–Wallis, respectively. The significance level was set at 5%.

## 4. Conclusions

This work studied different physicochemical parameters of liquids that were activated by NTAPP on the antibacterial action and toxicity of these liquids, focusing on the production of a decontaminating and antiseptic agent against the pathogen *Escherichia coli*. Assuming that the plasma activation of different solutions can generate variations of reactive oxygen-nitrogen species (RONS), we sought to analyze the impact of various liquids at a varied pH scale (2.5–3.5), which were defined by the activation time, which could affect the *E. coli*, and to identify what would be the safe conditions for their use. An analysis based on UV-vis spectrophotometer readings identified different RONS concentrations for the PALs. High concentrations of RONS, such as HNO_2_ and hydrogen peroxide (H_2_O_2_), were identified in the PAWs with an increase in the low pH. The PAL-SAL solution probably produces HOCl species, but we obtained high concentrations of RONS species, such as HNO_2_ and H_2_O_2_. The PALs had a strong antimicrobial efficacy in short- and long-acting periods, with a remarkable antibacterial action against *E. coli* at adjustable, low pHs. Additionally, in a general trend, the plasma activation configuration shows the safe conditions for their use. Conducting research with different PALs expands the production of disinfecting agents against *E. coli* and enables their use in various biomedical, food, environmental, and industrial applications.

## Figures and Tables

**Table 1:** Physicochemical parameters of tap water, deionized water, distilled water, filtered water, and saline solution, before and after activation by plasma for 1.0, 5.0, 30.0, and 60.0 min.

Sample	Plasma Exposure Time (min)	pH (±0.2)	ORP (±5.0 mV)	TDS (±5.0 ppm)	σ (±5 μS/cm)
PAW-TAP	0.0	6.60	19	40	50
	1.0	5.56	83	50	70
	5.0	3.55	193	160	220
	30.0	3.06	221	330	470
	60.0	2.57	250	720	720
PAW-DI	0.0	5.10	106	10	10
	1.0	4.00	169	40	50
	5.0	3.30	208	170	240
	30.0	2.95	228	450	310
	60.0	2.47	239	560	800
PAW-DIS	0.0	6.10	55	10	10
	1.0	4.02	168	60	80
	5.0	3.41	202	130	190
	30.0	2.94	228	660	950
	60.0	2.42	243	810	1160
PAW-FIL	0.0	6.90	6	70	110
	1.0	4.94	60	80	115
	5.0	3.50	198	140	200
	30.0	3.00	225	340	500
	60.0	2.62	238	630	900
PAW-SAL	0.0	7.20	7	9320	13,420
	1.0	3.70	188	9930	14,120
	5.0	3.50	210	10,180	14,630
	30.0	2.79	237	12,900	18,460
	60.0	2.52	253	17,400	>20,000

Abbreviations: ORP, oxidation–reduction potential; TDS, total dissolved solids.

**Table 2:** Concentration of long-lived RONS (H_2_O_2_, HNO_2_, NO_2_^−^ and NO_3_^−^) generated on tap water, deionized water, distilled water, filtered water, and 0.9% saline which were activated by plasma after 1.0, 5.0, 30.0, and 60.0 min.

Sample	Exposure Time (min)	pH (±0.09)	H_2_O_2_ (mg/L)	HNO_2_ (mg/L)	NO_2_^−^ (mg/L)	NO_3_^−^ (mg/L)
PAW-TAP	1.0	5.56	15.0	0.2	25.0	24.0
	5.0	3.55	80.3	33.5	47.6	53.3
	30.0	3.06	127.3	106.0	48.6	120.4
	60.0	2.57	111.7	374.4	55.6	69.3
PAW-DI	1.0	4.00	9.3	0.1	9.2	7.3
	5.0	3.30	76.5	57.9	46.2	47.4
	30.0	2.95	82.0	128.4	45.8	106.3
	60.0	2.47	160.3	525.0	62.0	69.3
PAW-DIS	1.0	4.02	23.7	2.2	9.4	11.0
	5.0	3.41	65.1	20.5	21.0	82.1
	30.0	2.94	95.8	73.6	25.6	193.7
	60.0	2.42	86.6	201.3	21.2	94.8
PAW-FIL	1.0	4.94	15.0	0.7	24.7	23.6
	5.0	3.50	48.8	36.5	46.1	36.6
	30.0	3.00	100.4	105.9	42.3	111.3
	60.0	2.62	157.2	446.3	74.4	43.1
PAW-SAL	1.0	3.70	22.0	5.5	11.0	12.0
	5.0	3.50	58.1	27.1	38.6	78.9
	30.0	2.79	106.9	175.0	43.2	165.2
	60.0	2.52	140.5	432.7	57.3	113.0

## Data Availability

The data presented in this study are available from the corresponding author upon reasonable request.
